# Serum CA19-9 as a marker of circulating tumor cells in first reflux blood of colorectal cancer patients

**DOI:** 10.18632/oncotarget.18912

**Published:** 2017-07-01

**Authors:** Jia-Xing Zhao, Li-Ren Liu, Xiao-Yu Yang, Fang Liu, Zhong-Guo Zhang

**Affiliations:** ^1^ State Key Laboratory of Cellular Stress Biology, Innovation Center for Cell Biology, School of Life Sciences, Xiamen University, Xiamen 361002, China; ^2^ Department of Colorectal Cancer Oncological Surgery, Large-Scale Data Analysis Center of Cancer Precision Medicine, Cancer Hospital of Chinese Medical University, Liaoning Provincial Cancer Institute and Hospital, Shenyang 110042, China; ^3^ Department of Gastrointestinal Cancer Biology, National Clinical Research Center of Cancer, Tianjin Medical University Cancer Institute and Hospital, Tianjin 300060, China

**Keywords:** CRC, colorectal cancer, CTC, circulating tumor cell, peripheral blood

## Abstract

Circulating tumor cells (CTCs) are used for metastasis surveillance in cancer patients, but low detection rates limit their use in colorectal cancer (CRC). We investigated the distribution of CTCs in peripheral and portal blood of CRC patients, and analyzed the relationship between serum tumor CEA/CA19-9 markers and CTCs blood levels. CTC levels detected in first reflux/portal vein blood were higher than in peripheral blood, and liver reduced CTCs amount. CTCs-positive patients had increased serum CEA and CA 19-9 levels, and the CEA and CA 19-9 levels correlated with the CTCs levels. Even in non-metastatic CRC patients with barely detectable CTCs in peripheral blood, serum CA 19-9 levels correlated with the CTC levels in first reflux/portal vein blood. These results demonstrate that CTC detection in the first reflux vein/portal vein blood is more sensitive than in peripheral blood, suggesting that clinical diagnosis using the CellSearch System should be based on the CTC detection in first reflux vein blood due to the high detection rates. In addition, our results indicate that serum CA 19-9 levels may serve as a diagnostic marker for further evaluation of CTC levels in portal blood.

## INTRODUCTION

Colorectal cancer (CRC) is one of the most commonly diagnosed cancers and the leading cause of cancer death worldwide [1]. Metastasis is the leading cause of CRC-related mortality, and is responsible for about 90% of CRC patient deaths [2]. About 50% of CRC patients have synchronous (15%~20%) [3, 4] or metachronous liver metastases (20%~30%) [5]. Compared with the overall CRC 5-year survival rates 65% and the 10-year survival rates 58%, the 5-year relative survival rate in non-metastatic CRC patients (39% of cases) is about 90% [6]. When patients are diagnosed in late stages, with colorectal liver metastases (CRLM), 5-year progress-free survival (PFS) and 5-year overall survival (OS) rates dramatically decrease [7, 8]. The lethal factor of CRC-related prognosis are metastases, especially liver metastases.

Circulating tumor cells (CTCs) contribute to metastases by being released into the blood from primary tumors [9]. The Veridex CellSearch system is the only CTCs detection method approved by U.S. FDA and Chinese CFDA for clinical CTCs detection. The Veridex CellSearch system captures CTCs using magnetic beads coated with an epithelial cell adhesion molecular antibody (anti-EpCAM); the CTCs are then identified using cytokeratin (CK) 8/18/19 +/DAPI +/CD45– staining. Detection of CTCs using the CellSearch system has been used as a clinical marker for prostate cancer [10], metastatic breast cancer [11], and colon cancer [12]. However, the CTCs-positive rates using the CellSearch system are low. For example, the median CTCs count was 0 in 7.5 mL of peripheral blood of 413 metastatic CRC patients [12]. In addition, CTCs were barely detectable using the CellSearch System in non-metastatic CRC patients [13]. However, even with the low detection rates, CTC is still the strongest prognostic factor in non-metastatic CRC patients [14, 15], and Cellsearch Systems remains the only method for CTCs-detection approved by the US FDA and Chinese CFDA. Thus, a more sensitive and accurate CTCs detection method using the Cellsearch system is urgently needed for CRC patients, particularly for non-metastatic CRC patients [13].

In the past, CTCs have been isolated almost exclusively from peripheral blood. Thus, the low detection rate might have been caused by an uneven release of CTCs into circulation system, and uneven distribution. Indeed, in pancreatic cancer, studies have shown higher CTC numbers in portal vein blood than in peripheral blood [16, 17]. In addition, portal blood CTCs-positive patients had higher liver metastasis rate than CTCs-negative patients after 3-year follow-up [18]. Like pancreatic cancer liver metastasis, colorectal liver metastasis (CRLM), the most frequent CRC metastatic site, is through the portal vein [19]. Tumor drainage (mesenteric) blood and portal blood of CRC patients had higher rates and numbers of CTCs than peripheral blood [20]. Furthermore, the hepatic venous (HV) CTCs>3 were associated with shorter PFS and OS, but not peripheral (PV) CTCs in CRLM patients [21]. However, there have been few studies comparing CTCs detected in portal venous blood vs. peripheral blood in CRC patients, and the relationship between CTCs and clinicopathological serum CRC markers is not known.

In this study, we investigated the distribution of CTCs in peripheral and portal blood of CRC patients, and we analyzed the relationship between serum tumor markers and CTCs counts in peripheral and portal blood.

## RESULTS

### Study population

From December 2015 to January 2017, 101 patients were enrolled prospectively into the study. The patients were divided into three groups: un-paired non-metastatic CRC patients (UP, n = 77; 42 patients were analyzed by using peripheral blood and 35 patients were analyzed using first flux vein blood), paired CRLM patients (n = 14), and paired non-metastatic CRC patients (NM, n = 10). The clinicopathological features of the patients are listed in Tables 1 and 2.

**Table 1:** Clinical and pathological data of un-paired non-metastases (UP) and CRLM CRC patients

	UP patients				CRLM patients	
	Peripheral vein blood		First reflux vein blood		CRLM patients	
	Patients (n)	Percentage (%)	Patients (n)	Percentage (%)	Patients (n)	Percentage (%)
Age (years)						
≥ 40 to > 60	13	31%	9	26%	5	36%
≥ 60	29	69%	26	74%	9	64%
Sex						
Male	25	60%	24	69%	8	57%
Female	17	40%	11	31%	6	43%
Tumor Location						
Colon	21	50%	12	34%	7	50%
Rectum	21	50%	23	66%	7	50%
T Classification						
T1	3	7%	0	0%	0	0%
T2	5	11%	3	9%	1	7%
T3	14	32%	10	29%	2	14%
T4	20	50%	22	62%	11	79%
Histologic Differentiated						
Well	3	7%	2	6%	1	7%
Moderately	29	69%	18	51%	6	43%
Poorly	10	24%	15	43%	7	50%
Lymph Node Metastasis						
Negative	30	71%	19	54%	3	21%
Positive	12	29%	16	46%	11	79%
Preoperative Serum CEA (ng/ml)						
< 15	10	24%	6	17%	8	57%
≤ 15	32	76%	29	83%	6	43%
Preoperative Serum CA19-9 (U/ml)						
< 37	2	5%	6	17%	7	50%
≤ 37	40	95%	29	83%	7	50%

CEA: carcinoembryonic antigen

CA19-9: carbohydrate antigen 19-9

**Table 2:** Clinical and pathological data of paired non-metastasis (NM) CRC patients

	Patients (n)	Percentage (%)
Age (years)		
≥ 40 to > 60	5	50%
≥ 60	5	50%
Sex		
Male	3	30%
Female	7	70%
Tumor Location		
Colon	3	30%
Rectum	7	70%
T Classification		
T1	1	10%
T2	1	10%
T3	2	20%
T4	6	60%
Histologic Differentiated		
Well	1	10%
Moderately	2	20%
Poorly	7	70%
Lymph Node Metastasis		
Negative	5	50%
Positive	5	50%
Preoperative Serum CEA (ng/ml)		
< 15	1	10%
≤ 15	9	90%
Preoperative Serum CA19-9 (U/ml)		
< 37	3	30%
≤ 37	7	70%

CEA: carcinoembryonic antigen

CA19-9: carbohydrate antigen 19-9

### CTCs detection in first reflux vein blood is more sensitive than in peripheral blood in un-paired non-metastatic (UP) patients

**Figure 1:**
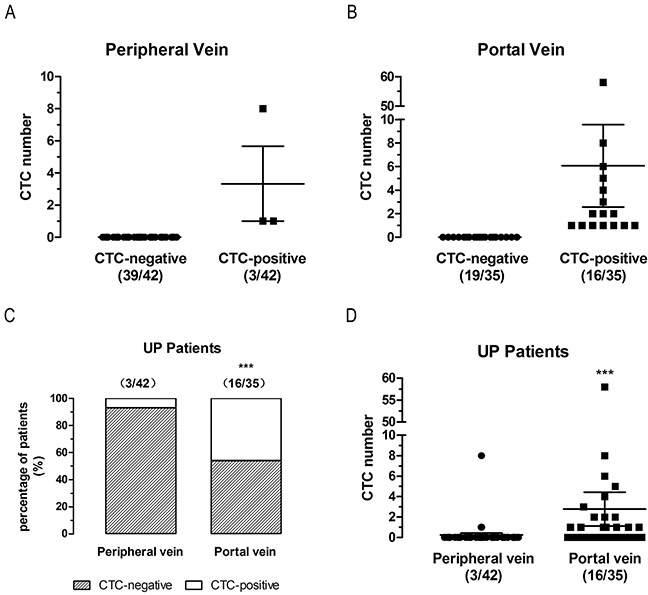
CTCs detection in first reflux vein blood is more sensitive than in peripheral blood in un-paired non-metastatic (UP) patients **(A)** CTCs number detected in peripheral vein blood. **(B)** CTCs number detected in first reflux vein blood. **(C)** Positive rates of CTCs-detected in peripheral and first reflux vein blood. **(D)** CTCs number detected in peripheral and first reflux vein blood.

42 CRC patients had peripheral vein blood collected preoperative for CTCs detection (Table 1). Consistent with previous studies, the CTCs number detected in peripheral blood of CRC patients by CellSearch system was low (Figure 1A). Only 7% patients had CTCs detectable (3/42).

**Figure 2:**
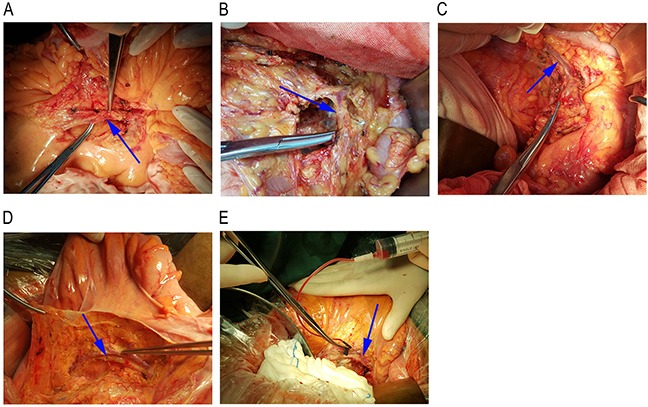
First reflux vein blood collection position First reflux vein blood was collected from the ileocolic vein of ascending or hepatic flexure **(A)**, the middle colon vein of transverse colon **(B)**, the left colon vein (upper or lower branch) of descending or splenic flexure **(C)**, the sigmoid vein of sigmoid colon cancer patients **(D)**, and the superior rectal vein of rectal cancer patients **(E)**.

We wanted to evaluate whether CTCs detection in portal vein was more sensitive. To avoid the influence of drainage blood from the superior mesenteric vein, splenic vein, and other reflux veins, we collected blood samples from the first branch vein belonging to the primary lesion. First reflux vein blood was collected from the ileocolic vein of ascending or hepatic flexure (Figure 2A), the middle colon vein of transverse colon (Figure 2B), the left colon vein (upper or lower branch) of descending or splenic flexure (Figure 2C), the sigmoid vein of sigmoid colon cancer patients (Figure 2D), and the superior rectal vein of rectal cancer patients (Figure 2E). The first reflux vein was isolated and blood sample was collected in bloodless dissection in order to prevent tumor cells and epithelial cells of tumor bed from contaminating circulation. Then, 10 mL of portal vein blood of 35 CRC patients was collected intraoperatively from the first reflux vein during CRC resection (Table 1). In addition, 7.5 ml of portal blood was analyzed for CTCs with CellSearch system.

The CTCs detection in first reflux vein blood (16/35) (Figure 1B) was more sensitive than in peripheral venous blood (3/42) (Figure 1A). Compared to 7% using peripheral vein blood, the CTCs detection rate in first reflux vein blood was 46% (P<0.0001, two-sided Fisher exact test, ORs=11.32) (Figure 1C). Consistent with the high CTCs-positive rate, CTCs in first reflux vein blood were detected at a significantly higher number than in peripheral vein blood (mean 2.77 vs 0.24, P<0.0001, two-tailed Mann Whitney test) (Figure 1D). These findings suggest that CTCs detection in first reflux vein blood is more sensitive than in peripheral vein blood in un-paired non-metastatic patients (UP).

### CTCs detection in first reflux vein blood is more sensitive than in peripheral blood in paired CRLM patients

As described above, CTCs detection in first reflux vein blood was more sensitive than in peripheral vein blood, but the un-paired test is not a conclusive evidence. Thus, peripheral blood and first reflux vein blood were analyzed in 14 paired colorectal cancer liver metastases (CRLM) patients (Table 1).

**Figure 3:**
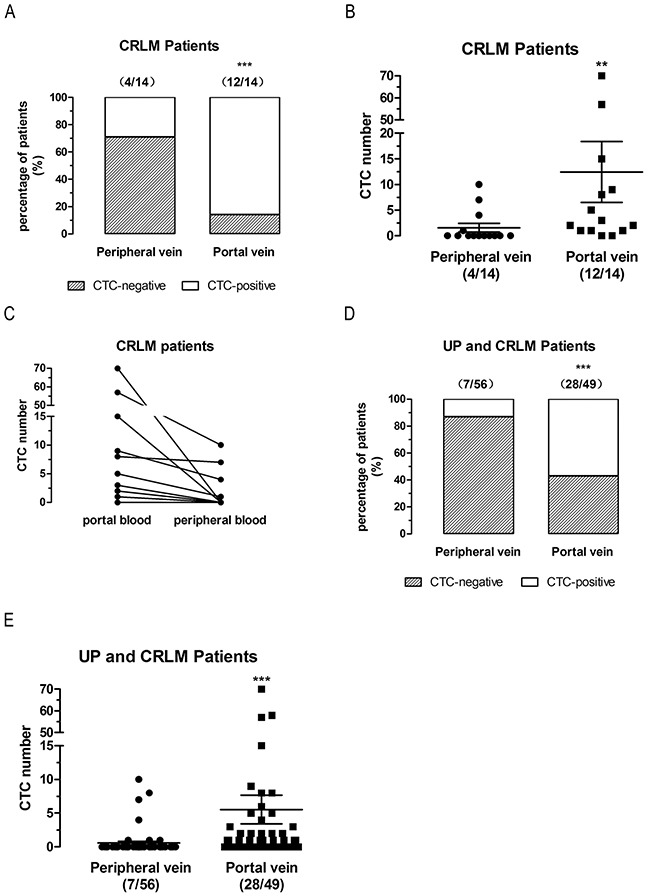
CTCs detection in first reflux vein blood is more sensitive than in peripheral blood in paired CRLM patients **(A)**, Positive rates of CTCs-detected in CRLM patients. **(B)**, CTCs number detected in CRLM patients. **(C)**, CTCs number variations in first reflux vein blood compared to peripheral vein blood of each CRLM patients. **(D)**, Positive rates of CTCs-detected in UP and CRLM patients. **(E)**, CTCs number detected in UP and CRLM patients. UP patients, un-paired non-metastatic patients; CRLM patients, colorectal liver metastatic patients.

CTCs were detected at a higher rate (12 [85.7%] vs 4 [28.6%], P<0.0001, two-sided Fisher’s exact test, ORs=15.04) (Figure 3A) and at a significantly higher number (mean 12.43 vs 1.57, P=0.0024, two-tailed Wilcoxon signed rank test) (Figure 3B) in first reflux vein blood than in peripheral venous blood of 14 CRLM patients. Next, we analyzed the CTCs counts change in peripheral blood and first reflux vein blood in the paired CRLM patients. The CTCs counts decreased in peripheral blood compared to first reflux vein blood in paired CRLM patients (Figure 3C).

Combined analysis of UP patients and CRLM patients also showed that CTCs detection in first reflux vein blood was more sensitive than in peripheral venous blood (28 [57%] vs 7 [13%], P<0.0001, two-sided Fisher’s exact test, ORs=8.871) (Figure 3D), with higher CTCs numbers (mean 5.53 vs 0.57, P<0.0001, two-tailed Mann Whitney test) (Figure 3E). These findings indicate that the CTCs detection in first reflux vein blood is more sensitive than in peripheral blood in CRLM and UP patients.

### CTCs detection in first reflux vein blood is more sensitive than in peripheral blood in paired non-metastatic (NM) patients

**Figure 4:**
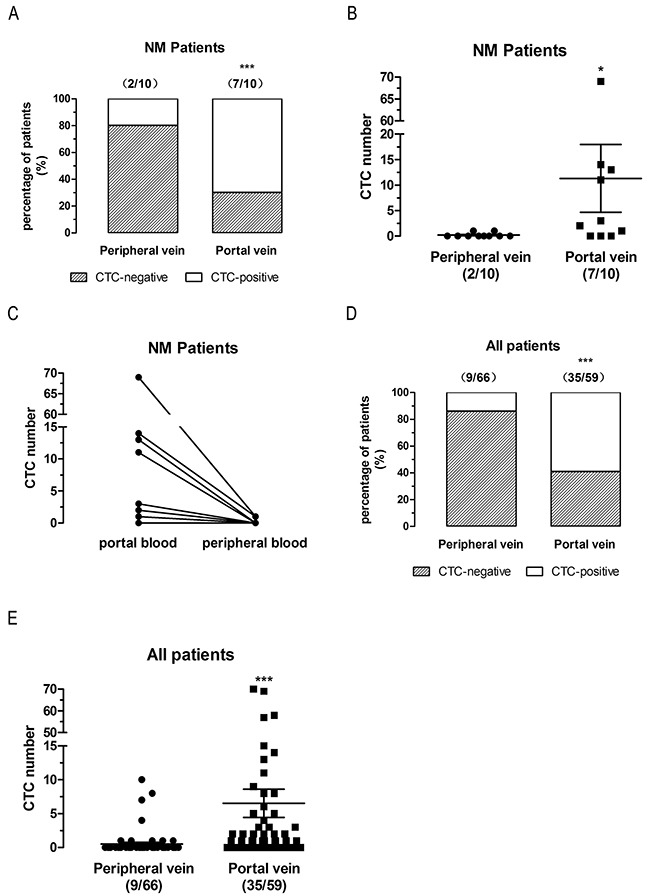
CTCs detection in first reflux vein blood is more sensitive than in peripheral blood in paired non-metastatic (NM) patients **(A)**, Positive rates of CTCs-detected in NM patients. **(B)**, CTCs number detected in NM patients. **C**, CTCs number variations in first reflux vein blood compared to peripheral vein blood of each NM patients. **(D)**, Positive rates of CTCs-detected in all patients. **(E)**, CTCs number detected in all patients. UP patients, un-paired non-metastatic patients; CRLM patients, colorectal liver metastatic patients; NM patients, paired non-metastatic patients.

To determine whether CTCs detection is more sensitive in first reflux vein blood of NM CRC patients than in peripheral blood, 10 NM patients were analyzed (Table 2). As expected, CTCs were detected at a higher rate (7 [70%] vs 2 [20%], P<0.0001, two-sided Fisher’s exact test, ORs=9.333) (Figure 4A) and a significantly higher number (mean 11.3 vs 0.2, P=0.0223, two-tailed Wilcoxon signed rank test) (Figure 4B) in first reflux vein blood than detected in peripheral venous blood. The CTCs counts variations in peripheral blood and first reflux vein blood in all paired NM patients were also analyzed. The results showed that the CTCs counts decreased in peripheral blood compared to first reflux vein blood (Figure 4C).

Combined analysis of CTCs levels in all three groups (UP, CRLM, and NM) also showed that CTCs detection in first reflux blood was more sensitive (35 [59%] vs 9 [14%], P<0.0001, two-sided Fisher’s exact test, ORs=8.840) (Figure 4D), and CTC levels (mean 6.51 vs 0.52, P<0.0001, two-tailed Mann Whitney test) (Figure 4E) were higher than in peripheral blood. These findings indicate that CTCs detection in first reflux vein blood is more sensitive than in peripheral blood in UP, CRLM, and NM patients, suggesting that clinical diagnosis using the CellSearch System should be based on the CTC detection in first reflux vein blood.

### CTCs amounts are not associated with primary cancer position or TNM stage in CRC patients

Because of the differences between colon and rectum cancer, we investigated whether CTCs in peripheral or portal blood were associated with primary cancer position. However, there was no statistical difference between colon and rectum cancer. In UP patients peripheral blood, only 1 of 21 colon cancer patients was CTCs-positive, and 2 of 21 in rectum cancer patients were CTCs-positive. In UP patients first reflux vein blood, 7 of 12 colon cancer patients were CTCs-positive, and 9 of 23 in rectum cancer patients were CTCs-positive. Similar results were also found in paired patients. In CRLM patients, 1 of 7 colon cancer patients and 3 of 7 rectum cancer patients were CTCs-positive in peripheral blood, and 6 of 7 colon cancer patients and 6 of 7 rectum cancer patients were CTCs-positive in first reflux vein blood. In NM patients, 1 of 3 colon cancer patients and 1 of 7 rectum cancer patients were CTCs-positive in peripheral blood, and 2 of 3 colon cancer patients and 5 of 7 rectum cancer patients were CTCs-positive in first reflux vein blood.

Next, we analyzed the relationship between CTCs and TNM stage. However, there was no statistical difference. Together, our results indicate that the amounts of CTCs are not associated with primary cancer position or TNM stage in CRC patients.

### High CEA/CA19-9 levels indicate high CTCs levels both in peripheral and first reflux vein blood in CRC patients

**Figure 5:**
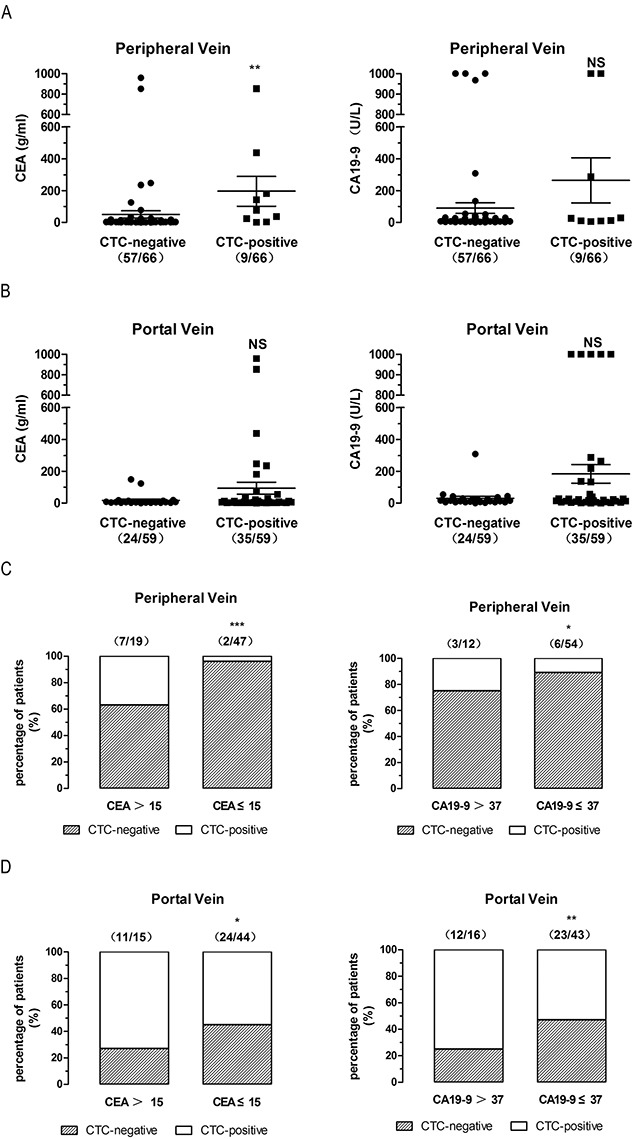
High CEA/CA19-9 levels indicate high CTCs levels both in peripheral and first reflux vein blood in CRC patients **(A)**, CEA/CA 19-9 levels of all patients with CTCs-detected in peripheral blood. **(B)**, CEA/CA 19-9 levels of all patients with CTCs-detected in first reflux/portal blood. **(C)**, CTCs-positive rates of different CEA/CA 19-9 levels in all patients with CTCs-detected in peripheral blood. **(D)**, CTCs-positive rates of different CEA /CA 19-9 levels in all patients with CTCs-detected in first reflux/portal blood.

To identify reliable and non-invasive CTC prognostic markers, we analyzed the relationship between CTCs and two traditional serum tumor markers, carcinoembryonic antigen (CEA) and carbohydrate antigen (CA) 19-9. Peripheral blood were collected preoperative for CEA and CA 19-9 analysis. In peripheral blood, all CTCs-patients had high CEA levels (mean 195.77 vs 50.11, P=0.0089, two-tailed Mann Whitney test) and high CA19-9 level (mean 264.74 vs 90.52, P=0.1325, two-tailed Mann Whitney test) (Figure 5A). Although CTCs were not correlated with CEA/CA19-9 levels in first reflux vein blood, CTCs-positive patients had high concentrations of CEA (mean 93.54 vs 17.90, P=0.5021, two-tailed Mann Whitney test) and CA19-9 (mean 183.43 vs 30.14, P=0.2766, two-tailed Mann Whitney test) (Figure 5B).

For clinical screening of CTCs-positive patients, we analyzed the relationship between patients with high CEA/CA19-9 and CTCs levels. In peripheral blood, the percentage of CTCs-positive patients was 37% in CEA>15 group, in contrast with 4% in CEA≤15 group (P<0.0001, two-sided Fisher’s exact test, ORs=0.07095) (Figure 5C). 19 out of 66 patients (29%) were CEA>15 with a sensitivity and specificity for CTC-positive of 22% and 79%, respectively. In addition, the percentage of CTCs-positive patients was 25% in CA19-9>37 group, compared to 11% in CA19-9≤37 group (P=0.0138, two-sided Fisher’s exact test, ORs=0.4158) (Figure 5C). 12 out of 66 patients (18%) were CA19-9>37 with a sensitivity and specificity for CTC-positive of 67% and 84%, respectively.

In first reflux vein blood, the percentage of CTCs-positive patients was 73% in CEA>15 group, compared to 55% in CEA≤15 group (P=0.0120, two-sided Fisher’s exact test, ORs=0.4521) (Figure 5D). 15 out of 59 patients (25%) were CEA>15 with a sensitivity and specificity for CTC-positive of 69% and 83%, respectively. The percentage of CTCs-positive patients was 75% in CA19-9>37 group, compared to 53% in CA19-9≤37 group (P=0.0019, two-sided Fisher’s exact test, ORs=0.3759) (Figure 5D). 16 out of 59 patients (27%) were CA19-9>37 with a sensitivity and specificity for CTC-positive of 66% and 83%, respectively. These results indicate that high CEA/CA19-9 levels might serve as a prognostic marker for high CTCs levels both in peripheral and first reflux vein blood in CRC patients.

### High CA19-9, but not CEA levels, indicate high CTCs levels in first reflux vein blood of non-metastatic patients

**Figure 6:**
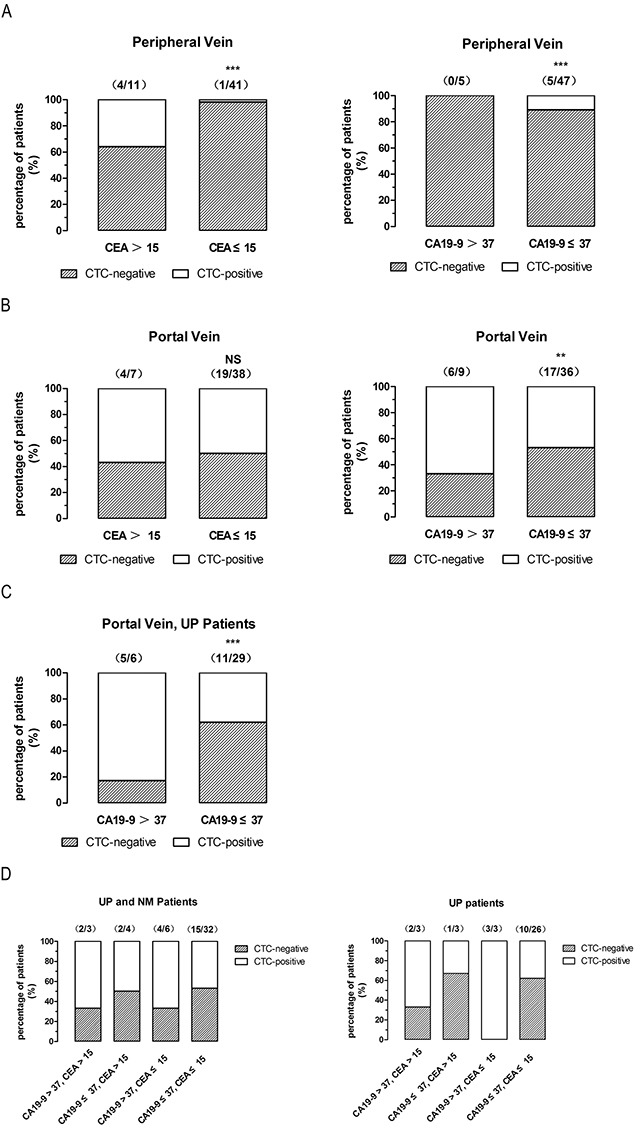
High CA19-9, but not CEA levels, indicate high CTCs levels in first reflux vein blood of non-metastatic patients **(A)**, CTCs-positive rates of different CEA/CA 19-9 levels in non-metastatic patients with CTCs-detected in peripheral blood. **(B)**, CTCs-positive rates of different CEA/CA 19-9 levels in non-metastatic patients with CTCs-detected in first reflux/portal blood. **(C)**, CTCs-positive rates of different CEA/CA 19-9 levels in UP patients with CTCs-detected in first reflux/portal blood. **(D)**, CTCs-positive rates of different CEA/CA 19-9 levels in non-metastatic/UP patients with CTCs-detected in first reflux/portal blood. UP patients, un-paired non-metastatic patients; NM patients, paired non-metastatic patients.

Since in non-metastatic colorectal cancer, CTCs are barely detectable in peripheral blood using the CellSearch System [13], we analyzed CTCs and CEA/CA19-9 levels in non-metastatic CRC patients (UP and NM patients). In peripheral blood of UP and NM patients, the percentage of CTCs-positive patients was 36% in CEA>15 group compared to 2% in CEA≤15 group (P<0.0001, two-sided Fisher’s exact test, ORs=0.03628) (Figure 6A). 11 out of 52 patients (21%) were CEA>15 with a sensitivity and specificity for CTC-positive of 20% and 85%, respectively. However, the percentage of CTCs-positive patients was 0% in CA19-9>37 group compared to 11% in CA19-9≤37 group (P=0.0007, two-sided Fisher’s exact test, ORs=25.83) (Figure 6A). 5 out of 52 patients (10%) were CA19-9>37 with a sensitivity and specificity for CTC-positive of 100% and 89%, respectively.

In first reflux vein blood, the percentage of CTCs-positive patients was 67% in CA19-9>37 group compared to 47% in CA19-9≤37 group (P=0.0065, two-sided Fisher’s exact test, ORs=0.7544) (Figure 6B). 9 out of 45 patients (20%) were CA19-9>37 with a sensitivity and specificity for CTC-positive of 74% and 86%, respectively. The percentage of CTCs-positive patients did not significantly change in the CEA>15 group (57%) and in the CEA≤15 group (50%) (P=0.3950, two-sided Fisher’s exact test, ORs=0.4368) (Figure 6B). 7 out of 45 patients (16%) were CEA>15 with a sensitivity and specificity for CTC-positive of 83% and 86%, respectively. Since most NM patients were T4 (6/10), UP patients were analyzed without NM patients. In first reflux vein blood, the percentage of CTCs-positive patients was 83% in CA19-9>37 group compared to 38% in CA19-9≤37 group (P<0.0001, two-sided Fisher’s exact test, ORs=0.1696) (Figure 6C). 6 out of 35 patients (17%) were CA19-9>37 with a sensitivity and specificity for CTC-positive of 69% and 95%, respectively. CTCs-positive patients had increased CA19-9 levels regardless of CEA levels (Figure 6D), suggesting that only the CA19-9 levels could serve as a potential marker for high CTCs in first reflux vein blood in non-metastatic CRC patients.

## DISCUSSION

In this study, we compared CTCs levels in portal and peripheral blood in 101 Chinese CRC patients divided in three groups: un-paired non-metastatic CRC patients (UP, n = 77), paired CRLM patients (n = 14), and paired non-metastatic CRC patients (NM, n = 10). Consistent with previous studies, CTCs levels were significantly higher in portal vein than in peripheral blood in UP (Figure 1), CRLM (Figure 3), and NM (Figure 4) patients. Moreover, 57% of CRLM patients (Figure 3C) and 50% of NM patients (Figure 4C) had CTCs-positivity only in the portal vein. In CTCs-positive patients, 67% of CRLM patients (Figure 3C) and 71% of NM patients (Figure 4C) had CTCs-positivity only in the portal vein. These patients would be missed by analyzing only peripheral blood. No patients were CTCs-positive in peripheral blood but negative in portal blood in paired CRLM patients (Figure 3C) and paired NM patients (Figure 4C). These findings provide new evidence that the CTCs-detection in first reflux vein blood is more sensitive than in peripheral blood in UP, CRLM, and NM patients. Further analysis revealed that the CTC counts decreased in peripheral blood compared to first flux vein blood in CRLM (Figure 3C) and NM patients (Figure 4C). Our results also provided new direct evidence for liver reduced CTCs amount. These results suggest that the CTCs levels in CRC patients should be analyzed in first reflux vein/portal vein blood, rather than in peripheral blood.

The high sensitivity of CTCs detection in first reflux vein blood might be attributed to two factors: First, CTCs from primary tumor site are released into first reflux vein/portal vein as “seed of metastases” [19], resulting in increased CTCs levels in first reflux vein/portal vein. Second, liver, as the unique organ blocking portal blood flux into peripheral vein, may serve as a goalkeeper or filter of CTCs released into peripheral vein [22]. However, serum liver markers AST/ALT were not different between CRLM patients and non-metastatic CRC patients (data not shown). Due to the small sample size and the low accuracy of AST/ALT assay, the relationship between liver lesions and CTCs in peripheral blood should be confirmed using more paired patients and better molecular markers of liver lesion.

A recent study has revealed that high CTCs counts in portal, but not in peripheral blood, are a significant prognostic predictor for liver metastases and DFS/OS [21]. However, CTCs in portal blood have few applications in clinical practice since portal blood samples cannot be easily obtained before surgery, and the clinical CTCs detection is still expensive. To develop a non-invasive, reliable, and affordable CTCs marker, we analyzed the relationship between traditional serum tumor markers and CTCs in CRC patients.

Serum tumor markers, such as carcinoembryonic antigen (CEA) and carbohydrate antigen (CA) 19-9 are widely used for cancer detection in clinical practice [23, 24]. CEA and CA 19-9 have a prognostic role in several cancers, including gastric, pancreatic, bile duct, bladder cancer, and CRC [25–29]. High serum CEA levels correlate with CRC patients’ prognosis [30, 31]. As recommended by the American Society of Clinical Oncology, CEA levels should be measured after curative surgery for recurrence surveillance in patients with stage II and III CRC [32–34]. Previous studies have demonstrated that the preoperative serum CA 19-9 level is a prognostic indicator in CRC patients [35–37]. CA 19-9 correlates with tumor cell-induced platelet aggregation [38], and adhesion of tumor cells to the endothelial cells of blood vessels [39], thus contributing to the distant metastases of CRC. In pancreatic cancer, CTCs-positive patients had higher CEA [18] and CA 19-9 [40] levels than CTCs-negative patients. Increased serum levels of CEA and CA19-9 were associated with detection of CTCs in peripheral blood of stage IV CRC patients [20]. Preoperative CEA and CA 19-9 levels were associated with CTCs-positive patients in peripheral blood, and shortened PFS/OS in CRC patients [14]. High tumor burden in the liver and high baseline serum CEA levels were associated with high CTCs in stage IV CRC patients [41]. However, there were no studies investigating the possible relationship between portal blood CTCs and serum tumor markers CEA/CA19-9 in CRC patients’ peripheral blood.

We found that CTCs-positive patients had high CEA/CA19-9 levels both in peripheral blood (Figure 5A) and in first flux vein blood (Figure 5B), and high CEA/CA19-9 patients had higher CTCs-positive percentage in peripheral blood (Figure 5C) and in first flux vein blood (Figure 5D). Our results indicate that high CA19-9 levels in peripheral blood may be used as a marker of CTCs in portal blood of CRC patients. Furthermore, CTCs-positive patients had increased CA19-9 levels regardless of CEA levels (Figure 6D), suggesting that only the CA19-9 levels may serve as a potential marker for high CTCs levels in non-metastatic CRC patients.

Our results show that CTCs detection in first reflux vein/portal vein blood is more sensitive than in peripheral blood. Furthermore, our results suggest that high CA 19-9 levels may be an early marker for selecting patients for CTC analysis in portal blood. These findings open a new path for CTCs detection in CRC patients. In the clinic, analysis of CTCs in peripheral blood should be avoided in CRC patients due to the low detectable rate and expensive cost. CRC patients with high CA 19-9 levels should be tested for CTCs levels in first reflux blood. The inexpensive and convenient traditional serum testing of CA 19-9 levels may signal CTCs in first reflux/portal vein blood.

The chief limitation of our study is the small sample size. In future, we want to investigate the correlation of portal CTCs-positive patients with liver metastases, recurrence-free survival, and overall survival. The mechanisms of how liver removes most of CTCs from portal vein blood should be investigated in future studies. A large-scale study with a subgroup analysis is also needed to confirm that peripheral serum tumor markers are related to portal CTCs and liver metastases.

## MATERIALS AND METHODS

### Patients’ recruitment

This single-institution study prospectively recruited patients with the following criteria: confirmed diagnosis of colorectal cancer with different stages, completed clinical and pathological results, and included biochemical test results. Patients who received any cancer-related treatment (including blood transfusion, preoperative radio-chemotherapy, or immunotherapy) within 1 month before blood sample collection and tumor detection were excluded, as well as those who underwent emergency surgery or surgery for recurrent disease. Furthermore, we excluded patients with a history of another malignancy that was diagnosed or treated within the past 5 years.

Patients who underwent surgical resection for histologically confirmed CRC at the Department of Colorectal Cancer Oncological Surgery, Large-scale Data Analysis Center of Cancer Precision Medicine, Liaoning Provincial Cancer Hospital & Institute (Cancer Hospital of China Medical University) from Dec. 2015 to Jan. 2017 were eligible for inclusion in this prospective study. Patients were recruited into three groups: un-paired non-metastatic CRC patients (UP group, n = 77; 42 patients were analyzed by using peripheral blood and 35 patients were analyzed using portal blood), paired CRLM patients (CRLM group, n = 14), and paired non-metastatic CRC patients (NM group, n = 10).

Enrollment of all patients in this study was approved by the Ethics Committee. Written informed consent was obtained and signed by all patients prior to sample collection.

### Clinical and pathological data recording

Clinical and pathological data were analyzed by reviewing electronic records (including primary tumor pathological characteristics). CEA and CA19-9 serum levels were analyzed using Roche Elecsys 2010 system. Imaging diagnosis of liver or other organ metastases were conducted in a multidisciplinary conference. Clinical TNM stage of patients was in accordance with the criteria of AJCC^7th^.

### Blood sample collection and CTCs counting

CRC patients had withdrawn 7.5 mL of blood from forearm peripheral vein and/or first reflux vein (portal vein) before surgery. Collected blood samples were immediately transferred to CellSave^®^ preservative tubes (Janssen Diagnostics, LLC, Raritan, NJ, USA) and analyzed within 3 days using Cellsearch System according to the standard CellSave^®^ protocol and the CTC Epithelial Cell Kit (Veridex). The first reflux vein was isolated and blood sample was collected in bloodless dissection in order to prevent tumor cells and epithelial cells of tumor bed from contaminating circulation.

### Statistical methods

All statistical analyses were performed with the GraphPad Prism 5.0 software. Values are expressed as the means ± SEM. The Two-sided Fisher’s exact test was used to compare ratios, and continuous variables were analyzed using Two-tailed Wilcoxon’s signed-rank test for paired patients or Two-tailed Mann Whitney test for un-paired patients. A probability value (*p*) < 0.05 was considered significant. The sensitivity and specificity calculations were performed using IBM SPSS Statistics version 19.0.0 (IBM Corporation, NY).

## Abbreviations

CRC: colorectal cancer
CTC: circulating tumor cell
UP patients: un-paired non-metastatic CRC patients
CRLM patients: colorectal liver metastatic patients
NM patients: paired non-metastatic CRC patients
CEA: carcinoembryonic antigen
CA 19-9: carbohydrate antigen 19-9
